# Molecular evidence of synaptic pathology in the CA1 region in schizophrenia

**DOI:** 10.1038/npjschz.2016.22

**Published:** 2016-06-29

**Authors:** Natalie Matosin, Francesca Fernandez-Enright, Jeremy S Lum, Martin Engel, Jessica L Andrews, Nils C Gassen, Klaus V Wagner, Mathias V Schmidt, Kelly A Newell

**Affiliations:** 1 Departments of Translational Research in Psychiatry and Stress Neurobiology and Neurogenetics, Max Planck Institute of Psychiatry, Munich, Germany; 2 Departments of Science, Medicine and Health, and Social Sciences, Illawarra Health and Medical Research Institute, University of Wollongong, Wollongong, NSW, Australia; 3 Department of Medicine, School of Psychiatry, University of New South Wales, Sydney, NSW, Australia; 4 Department of Health Science, School of Science, Australian Catholic University, Brisbane, QLD, Australia

## Abstract

Alterations of postsynaptic density (PSD)95-complex proteins in schizophrenia ostensibly induce deficits in synaptic plasticity, the molecular process underlying cognitive functions. Although some PSD95-complex proteins have been previously examined in the hippocampus in schizophrenia, the status of other equally important molecules is unclear. This is especially true in the cornu ammonis (CA)1 hippocampal subfield, a region that is critically involved in the pathophysiology of the illness. We thus performed a quantitative immunoblot experiment to examine PSD95 and several of its associated proteins in the CA1 region, using *post mortem* brain samples derived from schizophrenia subjects with age-, sex-, and *post mortem* interval-matched controls (*n*=20/group). Our results indicate a substantial reduction in PSD95 protein expression (−61.8%). Further analysis showed additional alterations to the scaffold protein Homer1 (Homer1a: +42.9%, Homer1b/c: −24.6%), with a twofold reduction in the ratio of Homer1b/c:Homer1a isoforms (*P*=0.011). Metabotropic glutamate receptor 1 (mGluR1) protein levels were significantly reduced (−32.7%), and Preso, a protein that supports interactions between Homer1 or PSD95 with mGluR1, was elevated (+83.3%). Significant reduction in synaptophysin (−27.8%) was also detected, which is a validated marker of synaptic density. These findings support the presence of extensive molecular abnormalities to PSD95 and several of its associated proteins in the CA1 region in schizophrenia, offering a small but significant step toward understanding how proteins in the PSD are altered in the schizophrenia brain, and their relevance to overall hippocampal and cognitive dysfunction in the illness.

## Introduction

The hippocampus is a region of the brain highly implicated in schizophrenia pathology, with reports from several laboratories consistently showing altered hippocampal form, volume, and function in the illness.^1^ This region, and especially the cornu ammonis (CA) sub regions, are crucial for cognitive functions that are weakened in individuals with schizophrenia.^2^ Healthy cognitive functions are largely dependent on dynamic changes in synapse configuration in the CA1 subfield, an important component of the hippocampus, which predominantly functions to consolidate inputs from CA3.^3^ This process of synaptic plasticity depends on the coordinated molecular activity of several proteins packed tightly into the postsynaptic density (PSD), with disruption to these molecules hypothesized to predispose neurons to synaptic deficits.^4^ Members of the PSD, particularly PSD95 and those with which it forms a complex, are thus of increasing interest for improving our understanding of the molecular processes underlying memory impairments and hippocampal pathology in schizophrenia.

PSD95 is the most abundant protein at the PSD. It is a member of the membrane-associated guanylate kinase family, a group of proteins that have prominent roles in synaptic plasticity.^5^ Consistent with this role, PSD95 is critical for molecular organization of the PSD^6^ and synapse stabilization.^7^ Previous studies have analyzed PSD95 expression in multiple regions of brains from patients with schizophrenia, although the results of these studies could be considered inconclusive; within many regions, the data are conflicting with either no change, increased, or decreased PSD95 mRNA or protein reported across cortical (e.g., dorsolateral prefrontal cortex) and subcortical (e.g., thalamus, nucleus accumbens, striatum) regions.^8–18^ Specifically within the hippocampus, studies have either detected increased PSD95 protein levels in the CA3 region of schizophrenia subjects,^19^ or no change in protein or transcript expression in CA1, CA4, the parahippocampal gyrus and dentate gyrus.^8,9,19–21^ Still, alterations of other important PSD95-complex scaffold proteins in CA1 that could contribute to synaptic pathology in schizophrenia cannot be excluded. For example, decreased levels of the critical scaffold protein Homer1 were reported in an unspecified region of the hippocampus,^22^ and increased levels of its related multi-scaffolding protein, Tamalin, was detected in CA1 in schizophrenia subjects.^23^ Preso proteins have a similar role as Tamalin;^24,25^ these proteins have been shown to be decreased in the dorsolateral prefrontal cortex in schizophrenia,^26^ but their status in the hippocampus in schizophrenia remains unknown.

In addition to its organizational role, PSD95 is one of the most stable proteins at excitatory glutamate synapses.^27,28^ PSD95 is a major regulator of protein–protein interactions, which is important for processes such as intracellular trafficking, cell-surface expression, recycling, and activity of ionotropic and group I metabotropic glutamate receptors (mGluRs), mGluR1 and mGluR5.^29,30^ These processes occur in concert with several other important scaffolding proteins, including Homer1 and Tamalin.^29–31^ Previously detected changes in Homer1 and Tamalin in the schizophrenia hippocampus^22,23^ might be related to alterations to glutamate transmission (specifically via mGluR activity), protein–protein interactions, trafficking, and gene expression, contributing to glutamate dysregulation which is apparent in the disorder. Indeed, recent studies have shown disruptions to NR2B subunit-containing *N*-methyl-*D*-aspartate receptors and α-amino-3-hydroxy-5-methyl-4-isoxazolepropionic acid receptors in the CA3 in schizophrenia,^19^ and accordingly, we recently found evidence of increased mGluR5 protein levels in the CA1 region of schizophrenia subjects relative to controls.^23^ It is unclear whether this finding extends to mGluR1, which has not been examined in the hippocampus in schizophrenia, although increased mGluR1a mRNA and protein levels have been previously reported in the prefrontal cortex of schizophrenia patients.^32,33^

Despite a recent report that disruptions to molecules in the PSD, such as PSD95 and NR2B subunit-containing *N*-methyl-*D*-aspartate receptors are specific to CA3 and not CA1 in schizophrenia,^19^ our previously reported alterations of mGluR5 and Tamalin in CA1^23^ suggest that disruptions to other important PSD95-complex molecules may exist in the CA1 region in schizophrenia. In addition, there is extensive evidence implicating CA1 in the illness; this includes reduced CA1 volume in first-episode schizophrenia patients,^34^ deformity of CA1 being related to symptom severity and antipsychotic response,^35^ and evidence that the CA1 subfield is hyperactive in the disorder.^36^ These studies point towards dysregulation of the molecules that comprise this region. We therefore aimed to explore the possibility that molecular abnormalities to important PSD95-complex proteins occur in CA1 by conducting a quantitative immunoblot study in a well-characterized *post mortem* brain cohort. We examined PSD95, Homer1 (short [Homer1a] and long [Homer1b/c] isoforms), Preso and mGluR1 proteins. Furthermore, levels of the presynaptic protein synaptophysin were assessed, as this protein has been shown to be a marker of synaptic density.^37^

## Results

### PSD95

**Figure 1:**
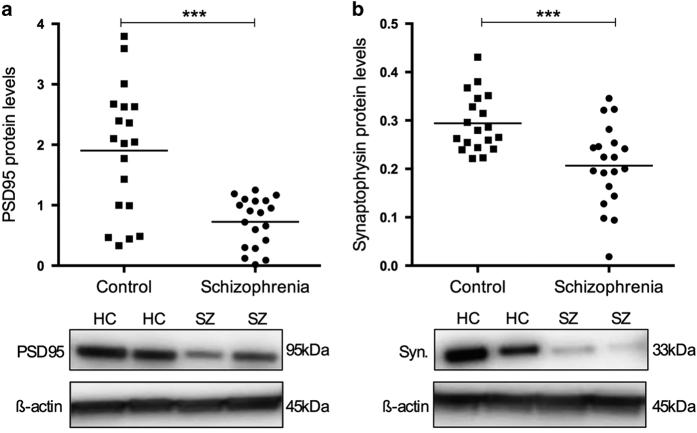
Scatterplots depicting normalized protein levels of (**a**) PSD95 and (**b**) synaptophysin in the CA1 region of healthy control (HC) and schizophrenia (SZ) subjects. Raw means (before co-varying the data) are depicted. ***P<0.001.

PSD95 is a major postsynaptic scaffold protein in the PSD, and aberrations in this protein can lead to deleterious effects on molecular synchronization in the PSD and synaptic plasticity.^6,7^ PSD95 was detected at the expected molecular weight of 95 kDa using immunoblot. Our results showed that PSD95 protein levels were significantly reduced in schizophrenia subjects compared with controls, after co-varying for brain pH and *post mortem* interval (−61.84%; F_1,34_=24.985, *P*<0.001; Figure 1a); these were variables associated with PSD95 protein levels (brain pH: *r*=−0.331, *P*=0.042 in all subjects and *r*=−0.542, *P*=0.017 in controls; *post mortem* interval: *r*=0.544, *P*=0.016 in schizophrenia subjects; Supplementary Table 1). There were no effects of hemisphere or illness duration on PSD95 protein measures, and no correlation with lifetime antipsychotic drug medication estimates. However a significant effect of antidepressant medication was seen for PSD95 (yes *n*=12; t_17_=3.289, *P*=0.005, −44.5%).

### Homer1a and Homer1b/c

**Figure 2:**
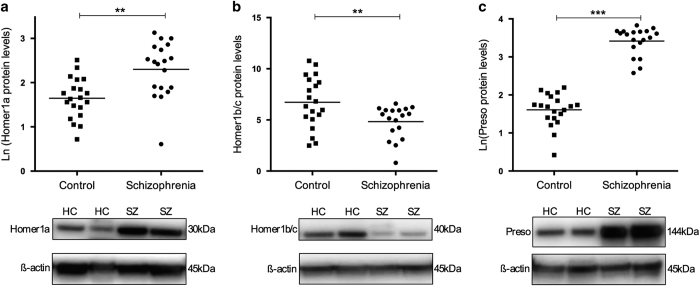
Scatterplots depicting normalized protein levels of (**a**) Homer1a, (**b**) Homer1b/c, and (**c**) Preso, in the CA1 region of healthy control (HC) and schizophrenia (SZ) subjects. Raw means (before co-varying the data) are depicted; Homer1a and Preso are depicted in the natural logarithm form owing to abnormal distribution of the data. **P<0.01, ***P<0.001.

Homer1 is a major scaffold protein of the PSD, which is known to have a role in the regulation of dendritic spine morphology and synaptic function in hippocampal neurons.^38^ To assess protein levels of Homer1 in the CA1 region in schizophrenia, the two main splice variants of Homer1 were measured using antibodies specific for Homer1a (short isoforms, an early immediate gene which acts as a dominant negative for the Homer1 long isoforms) and Homer1b/c (long isoforms, which are expressed constitutively). These proteins were detected at the expected molecular weights (Homer1a: 30 kDa, Homer1b/c: 40 kDa). A significant increase in protein levels of Homer1a was detected in schizophrenia subjects relative to controls, after co-varying for freezer storage time (+42.92%; F_1,34_=9.751, *P*=0.004; Figure 2a), which was a variable that correlated with Homer1a protein expression in control subjects only (*r*=0.545, *P*=0.013; Supplementary Table 1). Conversely, Homer1b/c levels were significantly reduced, after co-varying for freezer storage time (−24.55%, F_1,34_=4.810, *P*=0.035; Figure 2b), which correlated with Homer1b/c levels in all subjects (*r*=−0.341, *P*=0.039, Supplementary Table 1). These aberrations in Homer1a and Homer1b/c protein levels resulted in a significant twofold decrease in the ratio of Homer1b/c to Homer1a from 29.11±18.19 to 15.12±13.50 (t_35_=−2.679, *P*=0.011), suggesting an imbalance of these proteins in schizophrenia. There were no effects of hemisphere on Homer1 measures, and no correlation with lifetime antipsychotic drug medication estimates, or illness duration. However, a significant effect of antidepressant medication was seen on Homer1b/c levels (yes *n*=11; t_15_=2.950, *P*=0.010, −22.5%).

### Preso

Preso proteins facilitate PSD95/Homer1 long-isoform interactions with group I mGluRs, and reportedly have a role in dendritic spine morphogenesis.^24,25^ Considering the aforementioned alterations of PSD95 and Homer1, we additionally assessed protein levels of Preso in the CA1 region in schizophrenia. Preso was detected at the expected molecular weight of 144 kDa. Preso protein expression was significantly higher in schizophrenia subjects compared with controls (+83.30%; t_37_=14.142, *P*<0.001; Figure 2c). There were no effects of sample characteristics (age at death, pH, *post mortem* interval, RNA integrity number (RIN), brain weight, and freezer storage time) on Preso protein levels, or effects of hemisphere, antidepressant, or lifetime antipsychotic drug medication estimates. However, Preso protein levels were associated with age of disease onset in schizophrenia subjects (*r*=0.498, *P*=0.030).

### mGluR1

**Figure 3:**
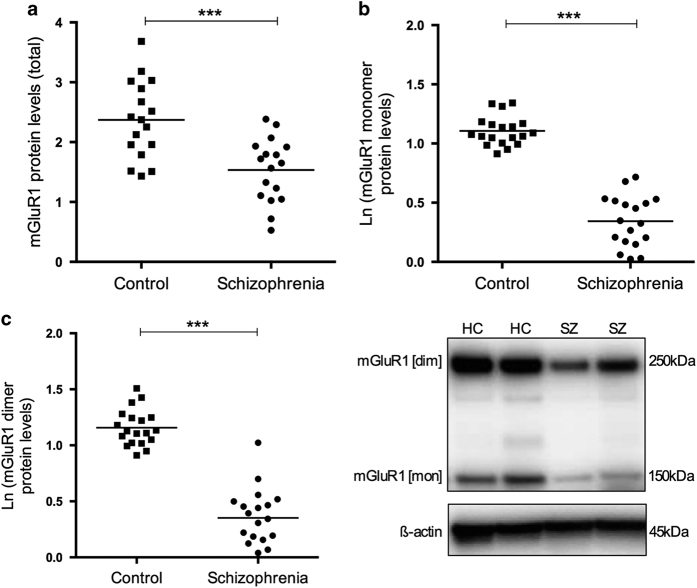
Scatterplots depicting normalized (**a**) total, (**b**) monomer, and (**c**) dimer measures of mGluR1 protein levels in the CA1 region of healthy control (HC) and schizophrenia (SZ) subjects. Raw means (before co-varying the data) are depicted; mGluR1 monomer and dimer levels are depicted in the natural logarithm form owing to abnormal distribution of the data. mGluR1 monomer levels were furher transformed (Ln[x]+1) to account for negative values. ***P<0.001.

mGluR1 is a key modulator of glutamatergic neurotransmission and synaptic plasticity, and its activity and trafficking is regulated by Homer1 and PSD95 via protein–protein interactions in the PSD.^30^ mGluR1 was detected at two molecular weights, thus monomer (150 kDa), dimer (250 kDa), and total (sum of monomer and dimer) levels are reported to assess protein levels of mGluR1. Total mGluR1 protein levels were significantly lower in schizophrenia subjects relative to controls maintained after co-varying for *post mortem* interval (−32.65%; F_1,30_=14.586, *P*=0.001; Figure 3a). Similarly, levels of mGluR1 monomer were reduced by −67.22% (F_1,33_=49.489, *P*<0.001, Figure 3b) and mGluR1 dimer by −66.41% (dimer: F_1,33_=60.885, *P*<0.001; Figure 3c), after also co-varying for *post mortem* interval, which was significantly correlated with all mGluR1 measures (Supplementary Table 1). There were no effects of hemisphere, illness duration, antidepressant, or lifetime antipsychotic drug medication estimates on mGluR1 proteins.

### Synaptophysin

Prior studies have shown that levels of synaptophysin are a reliable marker of synaptic density.^37^ Synaptophysin was thus also assessed using immunoblot, to determine whether the molecular alterations observed in the CA1 region in schizophrenia might extend to deficits in synapse numbers. Synaptophysin was detected at the expected molecular weight of 33 kDa. Protein levels of synaptophysin were significantly reduced in schizophrenia subjects compared with controls (−27.84%; t_36_=−3.558, *P*=0.001; Figure 1b). There were no effects of sample characteristics (age at death, pH, *post mortem* interval, RIN, brain weight, and freezer storage time) on synaptophysin protein levels, or effects of hemisphere, age of disease onset, antidepressant, or lifetime antipsychotic drug medication estimates.

### Protein–protein associations in schizophrenia relative to controls

To assess the hypothesis of abnormal protein–protein interactions at the synapse in schizophrenia, we ran exploratory analyses to assess the correlations between Group I mGluRs with these scaffold proteins (note that protein levels of mGluR5 and Tamalin were drawn from our previous work^13^). In controls, mGluR1 total levels were significantly correlated with PSD95 (*r*=0.612, *P*=0.012) and Homer1a (*r*=−0.654, *P*=0.004), whereas mGluR1 monomer levels were correlated with Homer1a (*r*=−0.612, *P*=0.005) and Homer1b/c (*r*=0.549, *P*=0.015). mGluR1 dimer correlated with PSD95 (*r*=0.530, *P*=0.020); all these associations were not detected in the schizophrenia group. mGluR1 monomer levels were significantly associated with Tamalin in control subjects (*r*=0.500, *P*=0.029) but not schizophrenia subjects, whereas mGluR1 total levels were associated with Tamalin in schizophrenia subjects (*r*=0.526, *P*=0.036), but did not reach significance in controls (*r*=0.463, *P*=0.061). No associations of mGluR5 and the scaffold proteins were observed in controls, although mGluR5 monomer and dimer levels were highly correlated with PSD95 (*r*>0.621, *P*<0.005) and Tamalin (*r*>0.611, *P*<0.005)^23^ in the schizophrenia group. A summary of these results are presented in Table 1, and the complete set of correlations for all other proteins measured in these subjects and regions are included in Supplementary Table 2.

## Discussion

This study shows extensive disruption to PSD95 and several important PSD95-complex proteins in *post mortem* samples from the CA1 hippocampal region of schizophrenia subjects relative to matched controls. In particular, substantial decreases in PSD95 and synaptophysin were observed, as well as increases and decreases in other related postsynaptic proteins suggesting altered protein–protein interactions in the illness. These data support the currently proposed model of hippocampal dysfunction in schizophrenia,^1^ centered on reduced volume and hyperactivity of the hippocampus which has been consistently reported in the illness.^36,39–43^

Prior mechanistic studies show that PSD95 is required to sustain molecular organization of the PSD^6^ and synapse stabilization.^7^ Deficiencies in PSD95 induced by RNA interference knockdown inflicts widespread deletions of entire segments of the PSD in rat hippocampal neurons,^6^ and impedes normal development of synapse structure and function after synaptic potentiation.^7^ The substantial reduction in PSD95 observed in this study (−62%) may therefore suggest the existence of deleterious effects to the PSD in the CA1 region in the illness. Similarly, synaptophysin proteins were reduced by almost 30% in the CA1 region of schizophrenia subjects compared to controls. Considering 95% of PSD95 protein is localized to synapses^44^ and the levels of synaptophysin and PSD95 strongly correspond to synapse numbers,^45,46^ these data are strongly suggestive of reduced synapse number in the CA1 region of schizophrenia subjects. To our knowledge, there are no studies examining possible changes to synapse density in the CA1 region of the schizophrenia brain, although reductions in gene expression of transcripts associated with dendritic spines has been reported in this region.^47^ In addition, increased spine density of pyramidal cell apical dendrites was reported in the adjacent CA3 region,^19^ which might be compensatory for synaptic alterations in CA1. An extension of this work in CA1 will be important to confirm the molecular findings of the current study and to determine on which synapses in CA1 these deficits are occurring (e.g., Schaffer collaterals), as well as how this fits with the findings in CA3.

The extensive aberrations in other PSD95-complex proteins in this study support the presence of altered synapses and plasticity in CA1 in schizophrenia. It is now established that Homer1 cooperates with PSD95 to form the architectural basis required for PSD receptor clustering and the appropriate signaling in response to prolonged neuronal stimuli.^48^ Upregulation of Homer1a proteins and downregulation of Homer1b/c were observed in this study, resulting in a twofold decrease in the ratio of Homer1b/c to Homer1a. The relative balance of Homer1b/c:Homer1a isoforms is considered to be functionally significant in the regulation of plasticity, dendritic spine morphology, and hippocampal-dependent cognitive function,^49^ with our data thus supporting the presence of synaptic dysregulation in CA1 in schizophrenia. Disruption of Homer1 function may also lead to reduced trafficking of group I mGluRs, their coupling to IP_3_ receptors and calcium homeostasis, which are processes required to appropriately direct plasticity.^50^ The Homer1b/c:1a ratio is described to act as a molecular switch that redirects signaling through either mGluR1 or mGluR5.^51,52^ mGluR1 protein levels were reduced in this study, suggesting that it has an inverse relationship with mGluR5, which are increased in the same region and subjects.^23^ In addition, Preso and Tamalin, which are proteins that facilitate PSD95/Homer1 long isoforms with mGluR1/5,^24,25,29,31^ were also upregulated, potentially in response to PSD95/Homer1 downregulation and an imbalance of mGluR1:mGluR5 levels. Preso proteins additionally direct synaptic plasticity by facilitation of the PSD95-complex to filamentous (F)-actin;^25^ overexpression of Preso might thus represent an attempt to reconnect PSD95 and F-actin molecules to repair deficits in plasticity.

A consistent finding in the hippocampus of schizophrenia patients—and CA1 in particular—is increased blood volume, supporting evidence of CA1 hyperactivity in the illness.^36,39–43^ Increased activity of CA1 is hypothesized to arise from deficient GABAergic firing in CA3, causing disinhibition of pyramidal cell inputs and increasing glutamate release onto CA1.^53^ Although it might be hypothesized that glutamate excitotoxicity leads to neuronal and thus volume loss in this region, the total number of neurons in the hippocampus is unaltered in schizophrenia.^54–57^ A recent immunostaining study however indicates reduced glutamic acid decarboxylase-immunoreactive neuropil density in *post mortem* schizophrenia hippocampal sections including CA1,^58^ which could contribute to hippocampal volume loss. In support, small deficits of interneuron populations such as parvalbumin- and somatostatin-positive interneurons have been reported in the hippocampus of schizophrenia subjects,^55,59–61^ ostensibly inducing hyperactivity via the disinhibition of pyramidal cells.^62^ It is interesting to note that the reduced levels of mGluR1 proteins in this study were detected with an antibody specific for the mGluR1α isoform, which is exclusively localized to interneurons of the CA1 region.^63,64^ Conversely, mGluR5, which is expressed on CA1 pyramidal neurons,^63^ was increased in the same region and subjects.^23^ The close correlation of mGluR1α and PSD95 seen in controls, but not schizophrenia subjects (Table 1), additionally suggests that the pathological reduction in PSD95 occurs alongside mGluR1α, which is consistent with the loss of interneurons observed in CA1.^55,59–61^

It should also be considered that our findings are in contrast to a recent report of unaltered PSD95 protein levels in the CA1 region in a similar sized schizophrenia *post mortem* cohort.^19^ Differences in age at death and manner of death may account for these inconsistent findings. However, considering the existence of a rostro-caudal gradient of gene expression in the hippocampus,^65,66^ it is plausible that small differences in dissection procedures and region collected in this study relative to the prior study^19^ have led to cohort-dependent differences and diverging results. Slight anatomical differences between samples may also explain the high levels of variability observed in control subjects in this study for PSD95 and Homer1b/c protein measures; this finding is opposite to typical observations in previous *post mortem* studies (i.e., greater spread of results in patients compared with controls). However, PSD95 and Homer1 proteins are also highly susceptible to activity- and experience-dependent change,^67,68^ especially in the CA1 region which is necessary for proper plastic processes. The spread of PSD95 and Homer1b/c:1a ratio may reflect a normal state, where these proteins are responding well to activity, experience and other stimuli. Alternatively, the lack of spread in schizophrenia subjects may represent an inability of patients to process or react to various stimuli, leading to altered experience- and activity-dependent molecular responses and ultimately disrupted plasticity.

A possible limitation of this study is the confounding effect of medication, as all schizophrenia subjects were medicated with antipsychotic drugs in this study. We did not observe any correlations with our protein measures and antipsychotic drug exposure. Our previous work additionally reports no influence of typical or atypical antipsychotic drug treatment on mGluR5, Preso and Tamalin protein expression in the hippocampus of rats.^23,69^ Others have shown that antipsychotic medication might influence transcript and protein expression of PSD95 and Homer1, but these were in regions other than the hippocampus.^70^ It may be of relevance that we also observed a non-significant borderline correlation between synaptophysin with lifetime antipsychotic drug exposure, considering the typical antipsychotic Trifluoperazine has been shown to reduce synaptophysin transcript expression within the Schaffer collateral region of CA1.^71^ Furthermore, chlorpromazine equivalents were used as an indicator of antipsychotic drug exposure in this study, which is an approach limited by the high rate of medication non-compliance among patients.^72^ Chlorpromazine equivalents are based on dopamine receptor D_2_ occupancy, which is relevant for typical antipsychotic medication history, but has limited value for patients who have a history of atypical antipsychotic drug exposure. It is also notable that an association of antidepressant drug exposure (yes/no) was observed with protein levels of Homer1b/c and PSD95. No data exists regarding the effects of antidepressant drug exposure on Homer1 or PSD95 proteins in the hippocampus, although Feyissa *et al.*^73^ reported no antidepressant effects in the anterior prefrontal cortex.

In summary, this study provides evidence of alterations to important PSD95-complex proteins in the CA1 region in subjects with schizophrenia. These included PSD95, Homer1 long and short isoforms, Preso, mGluR1, synaptophysin, as well as Tamalin^23^ and mGluR5^23^ proteins, which we have reported previously. Although the mechanisms and morphological effects of these alterations were not explored here, we speculate that there are changes to synaptic plasticity and/or neurophil loss in the CA1 region in schizophrenia, contributing to the increased activity and decreased volume of the hippocampus consistently observed in the disorder. Although the circumstantial support is persuasive these significant molecular leads require follow-up with morphological studies to confirm the existence of aberrant plasticity in the CA1 region in patients with schizophrenia.

## Materials and methods

### *Post mortem* brain samples

Human *post mortem* brain samples were from the NSW Brain Tissue Resource Centre (Sydney, NSW, Australia). Samples were derived from the CA1 region of 20 schizophrenia subjects (diagnosed according to the DSM-IV) and 20 controls (no history of psychiatric disorder), with these subjects matched according to *post mortem* interval, tissue pH, age at death, and RIN (Table 2); the sample was adequately powered (0.8–0.9) to detect expression differences (24%) owing to disease. Specimens were processed and characterized according to Weickert *et al.,*^74^ with samples in this study taken from the same subjects. Regarding dissection procedure, brain tissue was collected and dissected fresh at the level of the geniculate nucleus, and then frozen. From this level of dissection, architecture of the hippocampus could be visually verified and CA1 was manually dissected. When difficulty was encountered in identifying the hippocampal sub regions, tissue was further cryosectioned to confirm anatomical location. All subjects with schizophrenia were prescribed antipsychotics at the time of death and a lifetime chlorpromazine equivalent was calculated for each patient. This study was approved by the Human Research Ethics Committee at the University of Wollongong (HE99/222).

### Quantitative immunoblotting

Relative protein densities were determined by immunoblot analysis as described previously, with minor modifications to increase signal.^26^ Briefly, 5 μg of total protein was loaded per subject (blind to diagnosis) and run in duplicate. Probing was performed using the following primary antibodies: PSD95 (1:500, Millipore, Sydney, NSW, Australia, MAB1598); Homer1a (1:100, Santa Cruz, CA, USA, sc-8922), Homer1b/c (1:2000, ab97593, Abcam, Melbourne, VIC, Australia); Preso (1:1000, Santa Cruz, sc-242862); mGluR1 (1:250, Millipore, 07–617); synaptophysin (1:1000, Life Technology, Scoresby, VIC, Australia, 8H2L12 (monoclonal)). Blots were subsequently incubated with horseradish peroxidase-conjugated secondary antibody (anti-rabbit: 1:1000, Millipore, ap307P) and visualized using enhanced chemiluminescent detection kit (BioRad, Gladesville, NSW, Australia). Band densities were quantified with the Gel Doc 2200 Pro (Carestream Molecular Imaging, Woodbridge, CT, USA) and Carestream Molecular Imaging software (v5.0.4.44, Carestream Molecular Imaging). All proteins were within the linear range of detection for 5 μg protein (see Supplementary Information for details). Densitometry values for each sample were normalized to β-actin (1:5000; MAB1501, Millipore) by dividing protein values by β-actin values to account for differences in protein loadings between subjects; notably, β-actin was unaltered across cohorts.^23,75^ Values were subsequently normalized to a pooled sample (consisting of 10 pooled *post mortem* brain samples, with the same pool used across all experimental runs) to also account for potential gel-to-gel variability.

### Statistical analyses

Statistical analyses were performed with SPSS (Chicago, IL, USA, version 19). Significance was set to *P*<0.05 and data are presented as mean±s.e.m. The average of the duplicates for each protein measure was used for statistical analyses. PSD95, Homer1b/c, mGluR1 (total) and synaptophysin protein levels were normally distributed. mGluR1 (monomer) and mGluR1 (dimer) were skewed to the left, while Homer1a and Preso were skewed to the right (Kolmogorov–Smirnov: *d*=0.089–0.237; *P*<0.033); normalized distribution for these proteins was achieved by transforming to the natural logarithm of the relative protein values. Outliers were screened as mean±2 s.d. and removed. Two subjects were removed on average from each protein analysis.

Spearman’s correlations were implemented to analyze whether sample characteristics (age at death, pH, *post mortem* interval, RIN, brain weight, and freezer storage time) were associated with protein measures in all subjects and control and schizophrenia subjects individually. Where significant correlations were detected, analyses of covariance were performed to compare schizophrenia and control subjects, with significant correlates taken as covariates; where no significant correlations were detected, analysis was performed using independent *t*-tests. Two-way ANOVAs were used to determine main and interactive effects of diagnosis and brain hemisphere (left/right); gender analyses were not included owing to power constraints. In the schizophrenia group, lifetime antipsychotic drug history, age of disease onset, and duration of illness were also analyzed using Spearman’s correlations to extricate any potential effects on the data. Associations with antidepressant exposure (yes/no) with each of the protein levels were assessed using independent *t*-tests. Finally, the potential for differences in protein–protein associations between schizophrenia and controls groups were also assessed using Spearman’s correlations.

## Figures and Tables

**Table 1:** Relationships between mGluR1/5 and trafficking proteins in the CA1 region

	*Controls*						*Schizophrenia*					
	*Total*		*Monomer*		*Dimer*		*Total*		*Monomer*		*Dimer*	
*mGluR1*												
Homer1a	*r*=−0.654**	***P*****=0.004**	*r*=−0.612**	***P*****=0.005**	*r*=−0.429	*P*=0.059	*r*=−0.239	*P*=0.390	*r*=−0.071	*P*=0.795	*r*=−0.287	*P*=0.264
Homer1b/c	*r*=0.331	*P*=0.195	*r*=0.549*	***P*****=0.015**	*r*=0.361	*P*=0.118	*r*=0.218	*P*=0.455	*r*=0.064	*P*=0.820	*r*=0.153	*P*=0.572
PSD95	*r*=0.612*	***P*****=0.012**	*r*=0.465	*P*=0.052	*r*=0.530*	***P*****=0.020**	*r*=0.409	*P*=0.116	*r*=0.365	*P*=0.149	*r*=0.337	*P*=0.171
Tamalin	*r*=0.463	*P*=0.061	*r*=0.500*	***P*****=0.029**	*r*=0.376	*P*=0.102	*r*=0.526*	***P*****=0.036**	*r*=0.395	*P*=0.117	*r*=0.348	*P*=0.157
												
*mGluR5*												
Homer1a	*r*=−0.236	*P*=0.316	*r*=0.221	*P*=0.349	*r*=0.286	*P*=0.222	*r*=0.224	*P*=0.372	*r*=−0.09	*P*=0.723	*r*=−0.051	*P*=0.842
Homer1b/c	*r*=−0.116	*P*=0.627	*r*=0.253	*P*=0.283	*r*=0.060	*P*=0.801	*r*=0.191	*P*=0.462	*r*=0.422	*P*=0.092	*r*=0.414	*P*=0.098
PSD95	*r*=0.074	*P*=0.764	*r*=0.111	*P*=0.652	*r*=0.168	*P*=0.491	*r*=0.291	*P*=0.226	*r*=0.621**	***P*****=0.005**	*r*=0.716**	***P*****=0.001**
Tamalin	*r*=−0.029	*P*=0.905	*r*=0.334	*P*=0.150	*r*=0.292	*P*=0.212	*r*=0.132	*P*=0.591	*r*=0.611**	***P*****=0.005**	*r*=0.686**	***P*****=0.001**

Abbreviations: mGluR1/5, metabotropic glutamate receptor 1/5; PSD95, postsynaptic density protein 95; *r*, correlation coefficient.

Significant values are mentioned in bold font. *P<0.05; **P<0.01; ***P<0.001.

**Table 2:** Summary of *post mortem* subject demography

	*Control (*n*=20)*	*Schizophrenia (*n*=20)*
Brain pH	6.6±0.3	6.6±0.3
*Post mortem* interval (h)	26.1±12.8	28.3±10.1
RNA integrity number	7.2±0.7	7.2±0.5
Age at death (years)	58.2±12.6	55.5±13.5
Gender	2 F, 18 M	9 F, 11 M
Hemisphere	13 R, 7 L	10 R, 10 L
Age of disease onset (years)	—	23.5±6.8
Duration of illness (years)	—	32.05±13.7
Manner of death (natural/suicide)	20/0	16/4
Lifetime antipsychotic drug medication (standardized chlorpromazine equivalent, mg)	—	668±421
Antidepressant history (yes/no)	—	12

Abbreviations: F, female; L, left; M, male; R, right.

Data are expressed as mean±s.d.
