# Supplementary material for: Molecular evidence of synaptic pathology in the CA1 region in schizophrenia
Source: NPJ Schizophr. 2016 Jun 29;2:16022–. doi: 10.1038/npjschz.2016.22 (PMC4944906; doi:10.1038/npjschz.2016.22)
Supplement: Supplementary Information [file npjschz201622-s1.pdf]

Supplementary Table 1. Spearman's correlations of protein levels with demographic measures, disease characteristics and medication estimates.

(A) ALL		PSD95	Homer1 (a)	Homer1 (b/c)	Preso	mGluR1 (total)	mGluR1 (mon)	mGluR1 (dim)	Synaptophysin
AGE	Correlation Coefficient	0.171	0.173	0.132	0.02	0.178	0.105	0.171	0.266
	Sig. (2-tailed)	0.306	0.298	0.437	0.906	0.323	0.542	0.304	0.106
	N	38	38	37	39	33	36	38	38
RIN	Correlation Coefficient	0.006	-0.061	0.208	-0.097	-0.128	0.081	-0.081	-0.073
	Sig. (2-tailed)	0.969	0.714	0.217	0.555	0.478	0.64	0.628	0.665
	N	38	38	37	39	33	36	38	38
FST	Correlation Coefficient	-0.085	0.289	**-0.341***	0.18	-0.192	-0.225	-0.209	-0.045
	Sig. (2-tailed)	0.612	0.079	**0.039**	0.273	0.285	0.187	0.209	0.786
	N	38	38	**37**	39	33	36	38	38
pH	Correlation Coefficient	**-0.331***	-0.116	-0.054	-0.148	-0.022	0.058	-0.073	0.176
	Sig. (2-tailed)	**0.042**	0.489	0.753	0.368	0.904	0.738	0.663	0.289
	N	**38**	38	37	39	33	36	38	38
PMI	Correlation Coefficient	-0.142	0.013	-0.033	0.041	0.039	-0.048	-0.031	-0.097
	Sig. (2-tailed)	0.396	0.939	0.846	0.803	0.827	0.783	0.853	0.564
	N	38	38	37	39	33	36	38	38
BW	Correlation Coefficient	-0.016	-0.042	0.044	-0.153	0.147	0.15	0.151	0.055
	Sig. (2-tailed)	0.925	0.800	0.796	0.352	0.414	0.384	0.366	0.743
	N	38	38	37	39	33	36	38	38

(B) CONTROL		PSD95	Homer1 (a)	Homer1 (b/c)	Preso	mGluR1 (total)	mGluR1 (mon)	mGluR1 (dim)	Synaptophysin
AGE	Correlation Coefficient	0.389	0.008	0.422	0.084	0.27	0.167	0.259	0.176
	Sig. (2-tailed)	0.100	0.975	0.064	0.726	0.294	0.494	0.270	0.472
	N	19	20	20	20	17	19	20	19
RIN	Correlation Coefficient	0.048	0.337	0.231	0.121	-0.204	0.041	-0.144	-0.198
	Sig. (2-tailed)	0.844	0.146	0.326	0.610	0.432	0.867	0.545	0.417
	N	19	20	20	20	17	19	20	19
FST	Correlation Coefficient	-0.262	**0.545***	-0.247	0.035	-0.194	-0.196	-0.115	-0.211
	Sig. (2-tailed)	0.278	**0.013**	0.293	0.885	0.456	0.422	0.629	0.385
	N	19	20	20	20	17	19	20	19
pH	Correlation Coefficient	**-0.542***	-0.051	-0.309	-0.324	-0.27	0.018	-0.334	0.171
	Sig. (2-tailed)	**0.017**	0.830	0.186	0.163	0.295	0.943	0.150	0.483
	N	19	20	20	20	17	19	20	19
PMI	Correlation	-0.236	0.03	-0.22	-0.088	-0.284	-0.3	-0.214	0.106

	Coefficient								
	Sig. (2-tailed)	0.331	0.900	0.350	0.712	0.268	0.212	0.364	0.665
	N	19	20	20	20	17	19	20	19
BW	Correlation Coefficient	0.027	-0.139	-0.185	-0.373	0.299	0.063	-0.006	-0.091
	Sig. (2-tailed)	0.912	0.558	0.434	0.106	0.243	0.797	0.980	0.710
	N	19	20	20	20	17	19	20	19

**(C) SCHIZOPHRENIA**		**PSD95**	**Homer1 (a)**	**Homer1 (b/c)**	**Preso**	**mGluR1 (total)**	**mGluR1 (mon)**	**mGluR1 (dim)**	**Synaptophysin**
AGE	Correlation Coefficient	0.061	0.404	-0.227	0.197	0.25	0.198	0.179	0.331
	Sig. (2-tailed)	0.805	0.096	0.381	0.419	0.350	0.447	0.478	0.166
	N	19	18	17	19	16	17	18	19
RIN	Correlation Coefficient	-0.334	-0.415	-0.06	-0.391	-0.031	0.018	0.021	-0.207
	Sig. (2-tailed)	0.163	0.087	0.818	0.098	0.909	0.944	0.935	0.395
	N	19	18	17	19	16	17	18	19
FST	Correlation Coefficient	0.162	0.07	-0.148	0.155	-0.081	-0.271	-0.249	0.255
	Sig. (2-tailed)	0.506	0.782	0.570	0.528	0.766	0.293	0.319	0.291
	N	19	18	17	19	16	17	18	19
pH	Correlation Coefficient	-0.35	-0.31	0.151	-0.211	0.065	0.074	0.044	0.121
	Sig. (2-tailed)	0.142	0.211	0.563	0.387	0.812	0.779	0.861	0.621
	N	19	18	17	19	16	17	18	19
PMI	Correlation Coefficient	**0.544***	-0.174	0.472	-0.264	**0.534***	**0.598***	**0.521***	-0.126
	Sig. (2-tailed)	**0.016**	0.489	0.056	0.274	**0.033**	**0.011**	**0.026**	0.606
	N	**19**	18	17	19	**16**	**17**	**18**	19
BW	Correlation Coefficient	-0.262	0.297	0.34	0.397	-0.159	-0.104	-0.067	-0.137
	Sig. (2-tailed)	0.278	0.231	0.182	0.093	0.557	0.690	0.791	0.575
	N	19	18	17	19	16	17	18	19
AGE onset	Correlation Coefficient	0.082	-0.041	0.323	**0.498***	0.04	-0.141	0.11	0.203
	Sig. (2-tailed)	0.739	0.872	0.206	**0.030**	0.882	0.589	0.664	0.403
	N	19	18	17	**19**	16	17	18	19
Illness Duration	Correlation Coefficient	-0.006	0.444	-0.343	-0.035	0.25	0.222	0.159	0.196
	Sig. (2-tailed)	0.98	0.065	0.178	0.886	0.35	0.392	0.528	0.422
	N	19	18	17	19	16	17	18	19
APD Lifetime	Correlation Coefficient	-0.209	0.088	-0.309	-0.247	0.165	0.14	0.23	0.439
	Sig. (2-tailed)	0.391	0.729	0.228	0.307	0.542	0.593	0.358	0.060
	N	19	18	17	19	16	17	18	19

*Correlation is significant at the 0.05 level (2-tailed). **Correlation is significant at the 0.01 level (2-tailed). **Abbreviations** APD, antipsychotic drugs; BW, brain weight; dim, dimer; FST, freezer storage time; mon, monomer; PMI, postmortem interval; RIN, RNA integrity number.

Supplementary Table 2. Complete results of Spearman's correlations of protein-protein associations in (A) controls and (B) schizophrenia groups.

(A) Controls		Homer1a	Homer1bc	PSD95	Preso1	mGluR1tot	mGluR1mon	mGluR1dim	Synaptophysin	Tamalin	mGluR5tot	mGluR5mon	mGluR5dim
Homer1a	Correlation Coefficient	1	-0.356	-.518*	-0.072	-.654**	-.612**	-0.429	-.568*	-0.168	-0.236	0.221	0.286
	Sig. (2-tailed)	.	0.123	**0.023**	0.762	**0.004**	**0.005**	0.059	**0.011**	0.478	0.316	0.349	0.222
	N	20	20	19	20	17	19	20	19	20	20	20	20
Homer1bc	Correlation Coefficient	-0.356	1	.670**	0.379	0.331	.549*	0.361	0.195	0.43	-0.116	0.253	0.06
	Sig. (2-tailed)	0.123	.	**0.002**	0.099	0.195	**0.015**	0.118	0.424	0.058	0.627	0.283	0.801
	N	20	20	19	20	17	19	20	19	20	20	20	20
PSD95	Correlation Coefficient	-.518*	.670**	1	0.232	.612*	0.465	.530*	0.35	0.435	0.074	0.111	0.168
	Sig. (2-tailed)	**0.023**	**0.002**	.	0.34	**0.012**	0.052	**0.02**	0.155	0.063	0.764	0.652	0.491
	N	19	19	19	19	16	18	19	18	19	19	19	19
Preso1	Correlation Coefficient	-0.072	0.379	0.232	1	0.022	0.011	0.325	-0.118	0.383	0.188	0.365	0.275
	Sig. (2-tailed)	0.762	0.099	0.34	.	0.933	0.966	0.162	0.632	0.095	0.427	0.113	0.24
	N	20	20	19	20	17	19	20	19	20	20	20	20
mGluR1tot	Correlation Coefficient	-.654**	0.331	.612*	0.022	1	.812**	.919**	0.468	0.463	0.078	-0.123	0.005
	Sig. (2-tailed)	**0.004**	0.195	**0.012**	0.933	.	**0**	**0**	0.068	0.061	0.765	0.639	0.985
	N	17	17	16	17	17	16	17	16	17	17	17	17
mGluR1mon	Correlation Coefficient	-.612**	.549*	0.465	0.011	.812**	1	.577**	.587*	.500*	0.104	0.195	0.023
	Sig. (2-tailed)	**0.005**	**0.015**	0.052	0.966	0	.	**0.01**	**0.01**	**0.029**	0.673	0.424	0.926
	N	19	19	18	19	16	19	19	18	19	19	19	19
mGluR1dim	Correlation Coefficient	-0.429	0.361	.530*	0.325	.919**	.577**	1	0.412	0.376	0.029	0.183	0.274
	Sig. (2-tailed)	0.059	0.118	**0.02**	0.162	0	**0.01**	.	0.079	0.102	0.905	0.439	0.243
	N	20	20	19	20	17	19	20	19	20	20	20	20
Synaptophysin	Correlation Coefficient	-.568*	0.195	0.35	-0.118	0.468	.587*	0.412	1	0.3	0.104	-0.009	0.016
	Sig. (2-tailed)	**0.011**	0.424	0.155	0.632	0.068	**0.010**	0.079	.	0.212	0.673	0.972	0.949
	N	19	19	18	19	16	18	19	19	19	19	19	19
Tamalin	Correlation Coefficient	-0.168	0.43	0.435	0.383	0.463	.500*	0.376	0.3	1	-0.029	0.334	0.292
	Sig. (2-tailed)	0.478	0.058	0.063	0.095	0.061	**0.029**	0.102	0.212	.	0.905	0.15	0.212
	N	20	20	19	20	17	19	20	19	20	20	20	20
mGluR5tot	Correlation Coefficient	-0.236	-0.116	0.074	0.188	0.078	0.104	0.029	0.104	-0.029	1	-0.029	0.125
	Sig. (2-tailed)	0.316	0.627	0.764	0.427	0.765	0.673	0.905	0.673	0.905	.	0.905	0.6
	N	20	20	19	20	17	19	20	19	20	20	20	20
mGluR5mon	Correlation Coefficient	0.221	0.253	0.111	0.365	-0.123	0.195	0.183	-0.009	0.334	-0.029	1	.853**
	Sig. (2-tailed)	0.349	0.283	0.652	0.113	0.639	0.424	0.439	0.972	0.15	0.905	.	0
	N	20	20	19	20	17	19	20	19	20	20	20	20
mGluR5dim	Correlation Coefficient	0.286	0.06	0.168	0.275	0.005	0.023	0.274	0.016	0.292	0.125	.853**	1
	Sig. (2-tailed)	0.222	0.801	0.491	0.24	0.985	0.926	0.243	0.949	0.212	0.6	0	.
	N	20	20	19	20	17	19	20	19	20	20	20	20

(B) Schizophrenia		Homer1a	Homer1bc	PSD95	Preso1	mGluR1tot	mGluR1mon	mGluR1dim	Synaptophysin	Tamalin	mGluR5tot	mGluR5mon	mGluR5dim
Homer1a	Correlation Coefficient	1	-0.079	0.079	0.209	-0.239	-0.071	-0.287	-0.082	-0.018	0.224	-0.09	-0.051
	Sig. (2-tailed)	.	0.77	0.754	0.404	0.39	0.795	0.264	0.748	0.945	0.372	0.723	0.842
	N	18	16	18	18	15	16	17	18	18	18	18	18
Homer1bc	Correlation Coefficient	-0.079	1	.517*	0.118	0.218	0.064	0.153	-0.424	0.456	0.191	0.422	0.414
	Sig. (2-tailed)	0.77	.	0.034	0.653	0.455	0.82	0.572	0.09	0.066	0.462	0.092	0.098
	N	16	17	17	17	14	15	16	17	17	17	17	17
PSD95	Correlation Coefficient	0.079	.517*	1	0.007	0.409	0.365	0.337	-0.356	.809**	0.291	.621**	.716**
	Sig. (2-tailed)	0.754	**0.034**	.	0.977	0.116	0.149	0.171	0.135	0	0.226	**0.005**	**0.001**
	N	18	17	19	19	16	17	18	19	19	19	19	19
Preso1	Correlation Coefficient	0.209	0.118	0.007	1	-0.182	-0.262	-0.284	0.218	-0.046	0.393	0.068	-0.002
	Sig. (2-tailed)	0.404	0.653	0.977	.	0.499	0.309	0.254	0.371	0.853	0.096	0.781	0.994
	N	18	17	19	19	16	17	18	19	19	19	19	19
mGluR1tot	Correlation Coefficient	-0.239	0.218	0.409	-0.182	1	.959**	.953**	0.144	.526*	0.018	0.391	0.485
	Sig. (2-tailed)	0.39	0.455	0.116	0.499	.	0	0	0.594	**0.036**	0.948	0.134	0.057
	N	15	14	16	16	16	16	16	16	16	16	16	16
mGluR1mon	Correlation Coefficient	-0.071	0.064	0.365	-0.262	.959**	1	.931**	0.162	0.395	-0.167	0.174	0.275
	Sig. (2-tailed)	0.795	0.82	0.149	0.309	0	.	0	0.535	0.117	0.523	0.504	0.286
	N	16	15	17	17	16	17	17	17	17	17	17	17
mGluR1dim	Correlation Coefficient	-0.287	0.153	0.337	-0.284	.953**	.931**	1	0.222	0.348	-0.193	0.201	0.278
	Sig. (2-tailed)	0.264	0.572	0.171	0.254	0	0	.	0.376	0.157	0.443	0.423	0.265
	N	17	16	18	18	16	17	18	18	18	18	18	18
Synaptophysin	Correlation Coefficient	-0.082	-0.424	-0.356	0.218	0.144	0.162	0.222	1	-0.209	-0.154	-0.361	-0.298
	Sig. (2-tailed)	0.748	0.09	0.135	0.371	0.594	0.535	0.376	.	0.391	0.528	0.128	0.215
	N	18	17	19	19	16	17	18	19	19	19	19	19
Tamalin	Correlation Coefficient	-0.018	0.456	.809**	-0.046	.526*	0.395	0.348	-0.209	1	0.132	.611**	.686**
	Sig. (2-tailed)	0.945	0.066	0	0.853	**0.036**	0.117	0.157	0.391	.	0.591	**0.005**	**0.001**
	N	18	17	19	19	16	17	18	19	19	19	19	19
mGluR5tot	Correlation Coefficient	0.224	0.191	0.291	0.393	0.018	-0.167	-0.193	-0.154	0.132	1	0.435	0.439
	Sig. (2-tailed)	0.372	0.462	0.226	0.096	0.948	0.523	0.443	0.528	0.591	.	0.063	0.06
	N	18	17	19	19	16	17	18	19	19	19	19	19
mGluR5mon	Correlation Coefficient	-0.09	0.422	.621**	0.068	0.391	0.174	0.201	-0.361	.611**	0.435	1	.935**
	Sig. (2-tailed)	0.723	0.092	**0.005**	0.781	0.134	0.504	0.423	0.128	**0.005**	0.063	.	0
	N	18	17	19	19	16	17	18	19	19	19	19	19
mGluR5dim	Correlation Coefficient	-0.051	0.414	.716**	-0.002	0.485	0.275	0.278	-0.298	.686**	0.439	.935**	1
	Sig. (2-tailed)	0.842	0.098	**0.001**	0.994	0.057	0.286	0.265	0.215	**0.001**	0.06	0	.
	N	18	17	19	19	16	17	18	19	19	19	19	19

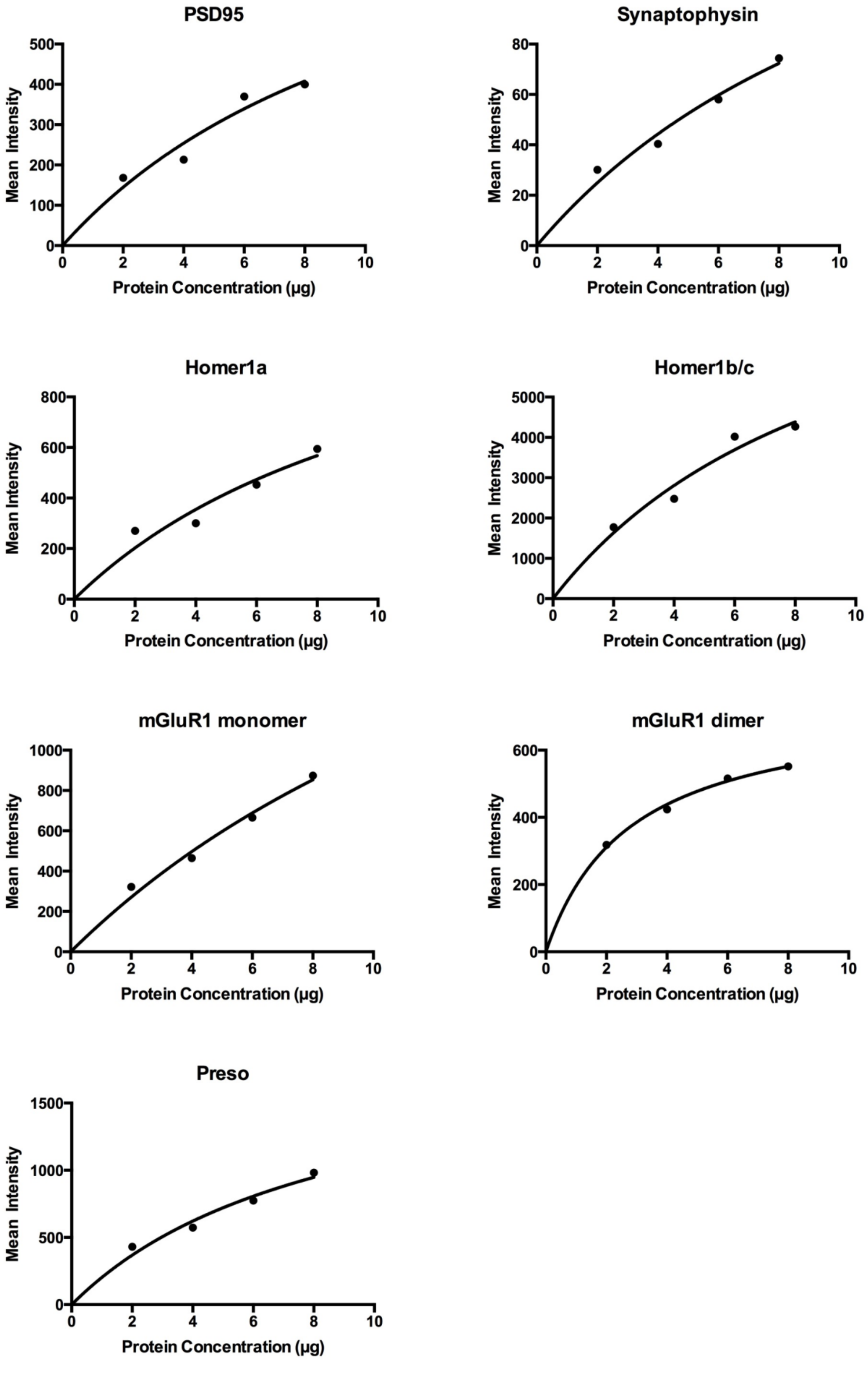

Supplementary Figure 1. Saturation curves determined by increasing protein concentrations versus mean optical intensity in the human hippocampal CA1 region.
